# Healthy diet associated with better asthma outcomes in elderly women of the French Asthma-E3N study

**DOI:** 10.1007/s00394-022-02815-0

**Published:** 2022-02-27

**Authors:** Wassila Ait-hadad, Annabelle Bédard, Sébastien Chanoine, Orianne Dumas, Nasser Laouali, Nicole Le Moual, Bénédicte Leynaert, Conor Macdonald, Valérie Siroux, Marie-Christine Boutron-Ruault, Raphaëlle Varraso

**Affiliations:** 1 Université Paris-Saclay, UVSQ, Univ. Paris-Sud, Inserm, Équipe d’Épidémiologie Respiratoire Intégrative, CESP, Villejuif, France; 2 Team of Environmental Epidemiology Applied To Reproduction and Respiratory Health, Institute for Advanced Biosciences (IAB), Inserm U1209, CNRS, University Grenoble Alpes, Grenoble, France; 3 Université Paris-Saclay, UVSQ, Univ. Paris-Sud, Inserm, Gustave Roussy, Équipe “Exposome Et Hérédité”, CESP, 94805 Villejuif, France

**Keywords:** Alternate Healthy Eating Index-2010 (AHEI-2010), Asthma symptom score, Asthma control, Metabolic multimorbidity-related medications, Body mass Index, Asthma prevention, Asthma management

## Abstract

**Purpose:**

The impact of a healthy diet on asthma prevention and management, particularly among elderly women, remains poorly understood. We investigated whether a healthy diet would be associated with fewer asthma symptoms, and, among women with asthma, with reduced uncontrolled asthma and metabolic-related multimorbidity.

**Methods:**

We included 12,991 elderly women (mean age = 63 years) from the Asthma-E3N study, a nested case–control study within the French E3N cohort. Negative binomial regressions were used to analyse associations between a healthy diet [evaluated by the Alternate Healthy Eating Index-2010 (AHEI-2010)] and a validated asthma symptom score, and logistic regressions to analyse associations between the AHEI-2010 with the asthma control test and multimorbidity profiles previously identified by clustering methods on medications used.

**Results:**

After adjustment for potential confounders, a linear inverse association was found between the AHEI-2010 score and the asthma symptom score [mean score ratio (95% CI) = 0.82 (0.75–0.90) for the highest versus lowest quintile; *p* for trend < 0.0001]. In addition, women in the highest versus lowest AHEI-2010 tertile were at a lower risk to belong to the “Predominantly metabolic multimorbidity-related medications profile” compared to the “Few multimorbidity-related medications" profile [OR 0.80 (0.63–1.00) for tertile 3; *p* for trend = 0.05; *n* = 3474].

**Conclusion:**

Our results show that a healthy dietary intake could play an important role in the prevention and management of asthma over the life course.

**Supplementary Information:**

The online version contains supplementary material available at 10.1007/s00394-022-02815-0.

## Introduction

As recently underlined in the Lancet [1], given the immense societal and individual burden of asthma, there is an urgent need to further develop novel strategies to limit the disease and its consequences. Asthma control, the main objective of asthma management, remains suboptimal in roughly one asthma patient out of two, with even higher rates among women [2, 3]. In this context, investigating the role of modifiable lifestyle factors such as diet is key for the primary and secondary prevention of this highly prevalent disease. While there are promising findings for possible dietary intervention to reduce asthma in children [4], the impact of diet on asthma in adults, and even more so among the elderly, remains largely unknown.

Among adults over 65 years, the prevalence of asthma varied from 4 to 13% [5], a rate likely underestimated as it is frequently underdiagnosed in this age group [6]. The burden of asthma is more significant in the elderly than in their younger counterparts [7, 8] with more hospitalisations [6, 8], and worse health-related quality of life [5, 6, 8] and asthma control [6]. Asthma in the elderly is a phenotype of interest, especially in women because asthma tends to be more prevalent (9.9% vs 6.2%) and more severe in women than in men [9]. Obesity is now an established risk factor for asthma [10, 11], with recent studies supporting the hypothesis that obesity is causally related to asthma [12], and with a higher risk in elderly women as compared to men [13]. Furthermore, multimorbidity is common in asthma patients, especially in the elderly [13]. Besides obesity, allergic rhinitis, chronic obstructive pulmonary disease (COPD), gastroesophageal reflux, and sleep apnoea syndrome that are the most common asthma-related multimorbidities, recent studies have suggested that other chronic conditions such as cardiovascular diseases (CVD), metabolic syndrome, or type 2 diabetes mellitus are also involved [14].

Although published studies on the diet–asthma association have traditionally focused on specific nutrients or foods, it is important in terms of dietary recommendations to emphasize overall dietary patterns rather than specific foods and nutrients to account for synergistic effects on health of foods and nutrients within the overall diet [15]. Several dietary scores have been proposed to globally assess diet quality and among them, the Alternate Healthy Eating Index-2010 (AHEI-2010) score, that reflects a healthy diet, has been associated with a lower risk of chronic diseases [16]. Among middle-aged women and men, a better adherence to a healthy diet evaluated by the AHEI-2010 was associated with a lower asthma symptom score [17, 18] and better asthma control [18]. Being a continuous measure of asthma, the asthma symptom score that has been associated with new onset of asthma, provides more power to detect risk factors for asthma as compared to a dichotomous definition [19]. Up to now, the role of a healthy diet on asthma symptoms, asthma control, and asthma-related metabolic and cardiovascular morbidities, which are commonly associated with both asthma [20] and an unhealthy diet [21], remains unknown among elderly women.

In a large study among elderly women, we aimed to investigate (1) the association between a healthy diet assessed with the AHEI-2010, and the asthma symptom score and, (2) among patients with asthma, the association of this dietary score with uncontrolled asthma and with specific metabolic and cardiovascular multimorbidity-related medications profile.

## Methods

### Study population

The E3N study (Etude Epidémiologique auprès des femmes de la Mutuelle Générale de l’Education Nationale [MGEN]) is a prospective cohort which was initiated in 1990 to investigate risk factors of major non-communicable diseases in among 98,997 women affiliated to the a French national health insurance plan covering mostly teachers [22]. Since 1990, information on lifestyle and medical history has been collected approximately every 2 years by means of self-administered questionnaires.

**Fig. 1:**
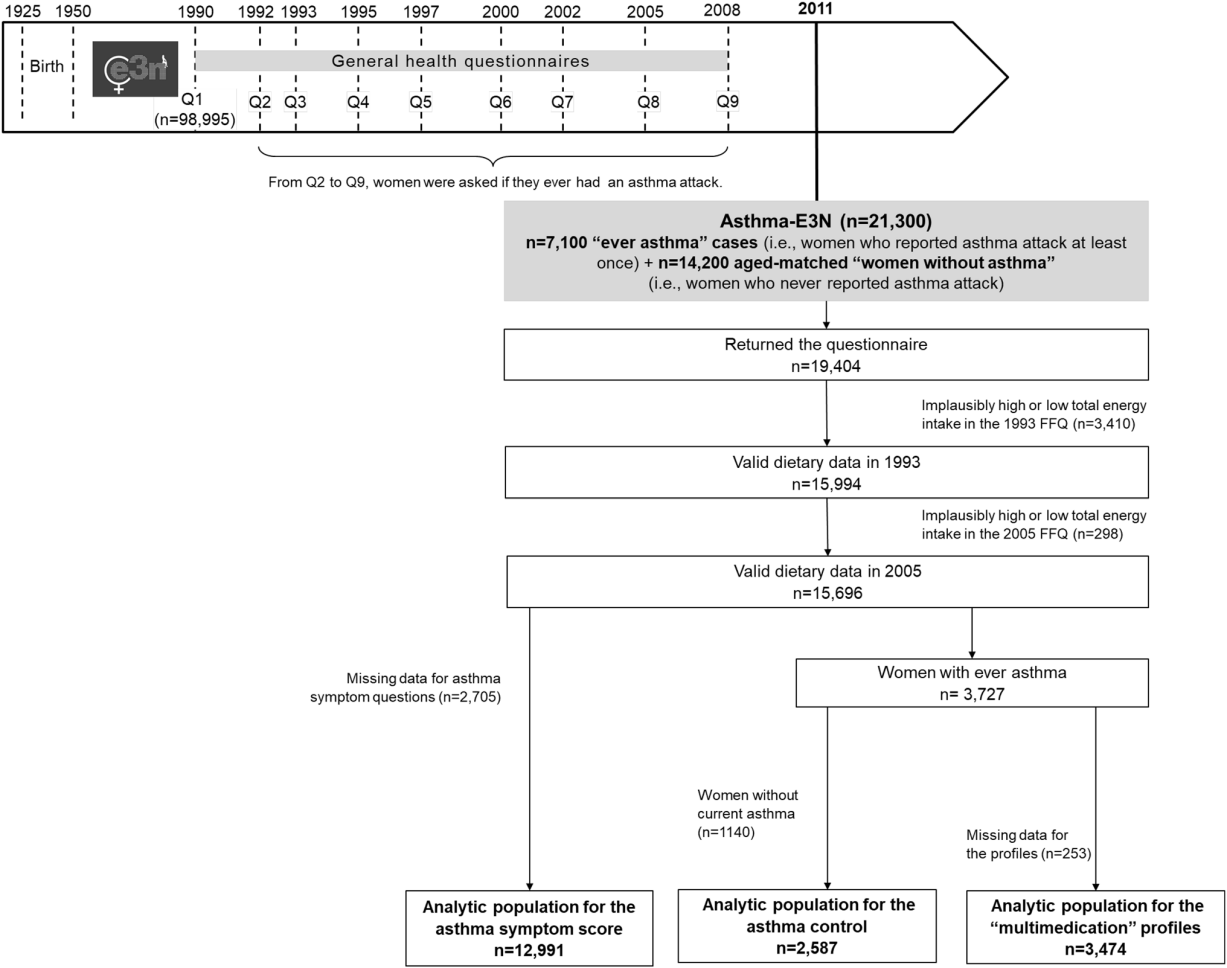
Study design and flow chart of participants

Current analyses were conducted on women participating in the Asthma-E3N study, a nested case–control study on asthma within the E3N study, conducted in 2011. Briefly, 7,100 cases, e.g., women who gave a positive answer to the single question “Have you ever had an asthma attack?” in the main questionnaires at least once between 1992 and 2008, and 14,200 age-matched women without asthma, i.e., women who never reported any asthma attack between 1992 and 2008. Out of the 21,300 women invited to participate to the Asthma-E3N study, 19,404 women responded to the respiratory health questionnaire (91% response rate) (Fig. 1). We excluded women who did not complete the food questionnaire in 1993 or in 2005, or had an implausibly high (top 1% of the ratio between energy intake and energy requirement (EIER)) or low (bottom 1% of the EIER ratio) total energy intake in 1993 or in 2005 (*n* = 3708). For the asthma symptom score, we further excluded women who did not answer to the asthma symptom questions (*n* = 2705). For “multimedication” profiles, we first selected women who reported “ever asthma” (*n* = 3474), and for asthma control, we further excluded women without current asthma (*n* = 1140). The analytic population included 12,991 women for the asthma symptom score, 2587 women for the asthma control, and 3474 women for the “multimedication” profiles.

Comparison of excluded vs included participants is presented in eTable 1. Excluded participants because of missing dietary data (*n* = 3708) were older than included participants (*n* = 12,991). After adjustment for age, excluded participants had a lower educational level, were less often married, and had more missing data for asthma outcomes, as compared to included women. When additionally excluding participants because of missing data for the asthma symptom score (*n* = 2705), excluded women were significantly older than included participants. After adjustment for age, excluded participants consumed slightly more energy intake, were less physically active, were more often current smokers, had a lower educational level, and were more often overweight or obese, as compared to included women.

The study protocol was approved by the French Institutional Ethics Committee, and all participants provided their written informed consents.

### Diet assessment

We calculated the AHEI-2010 from two validated semi-quantitative food history questionnaires administered in 1993 and 2005. The first part of the questionnaire was semi-quantitative and assessed consumption frequencies and portion sizes for the eight potential daily meals (breakfast, morning snack, aperitif before lunch, lunch, afternoon snack, pre-dinner aperitif, dinner, and after dinner snack) for 66 foods or food groups. Frequency was quantified in 11 potential categories: never or less than once a month; 1, 2, or 3 times a month, and 1 to 7 times a week. To facilitate the estimation of portion sizes, a photo booklet was also sent [23]. The second part of the questionnaire was qualitative and allowed detailing the consumption of specific foods within the food groups mentioned in the first part of the questionnaire.

The AHEI-2010 has been previously described [16]. It includes 11 items: 6 items considered as beneficial (vegetables, fruits, whole grains, nuts and legumes, long-chain Ω-3 fatty acids, and polyunsaturated fatty acids); 1 item for which moderate consumption is recommended (alcohol); and 4 items for which avoidance or low consumption is considered best (sugar-sweetened beverages and fruit juices, red/processed meat, trans fat, and sodium). In our study, we calculated a modified AHEI-2010 including all items except trans fat, which was not available. The total score ranged from 0 to 100, a higher score representing a healthier diet. When the sample size allowed it, we categorized the AHEI-2010 score in quintiles, otherwise in tertiles.

### Asthma outcome assessment

The asthma symptom score has been previously proposed as a validated continuous measure of asthma in epidemiological studies [24, 25]. It is particularly relevant in epidemiological studies, as it can be used in participants both with and without asthma allowing integration of different asthma phenotypes and different levels of asthma prevention. It ranges from 0 to 5 according to the presence or absence of five respiratory symptoms during the past 12 months.

To assess asthma control, we used the asthma control test (ACT), a validated self-administered questionnaire based on five questions on activity limitations, frequency of symptoms, and frequency of use of quick-relief medication in the past 4 weeks [26]. The total ACT score ranges from 5 to 25 and we categorised asthma as controlled (ACT > 19) versus uncontrolled (ACT ≤ 19) [26]. Due to the large sample size, we further distinguished participants with a score of 25 and participants with a score of 20–24, as previously done [27]. Asthma control was therefore classified as: controlled asthma (ACT score = 25), partly controlled asthma (ACT score between 20 and 24), and poorly controlled asthma (ACT score ≤ 19) [26].

Multimorbidity-related medication profiles in asthma were identified using latent class analysis (LCA) based on data from the exhaustive MGEN drug database, and personal characteristics (body mass index, sleep apnoea syndrome and smoking status), as previously described in detail [28]. Briefly, non-hospital-delivered drug classes were identified over the 2 years before the Asthma-E3N questionnaire from the exhaustive MGEN drug reimbursement database using the Anatomical Therapeutic Chemical (ATC) code. Latent class analyses (LCA), i.e. a data-driven approach, were performed to identify asthma groups characterised by specific multimorbidity-related medication profiles. Te following three profiles were identified: (1) the “Few multimorbidity-related medications” profile, (2) the “Predominantly allergic multimorbidity-related medications”, and (3) “Predominantly metabolic multimorbidity-related medications”.

### Covariates

Self-administered questionnaires were used to collect data on socio-demographic and lifestyle characteristics. Energy intake was computed in 1993 and 2005 using a Food Composition Database derived from the French Information Center on Food Quality (CIQUAL) and expressed in kilocalories/day (kcal/day) [29]. Physical activity was assessed in 2005 using data from several questions on different activities [30] and expressed in metabolic equivalent of tasks (METs) per week (MET-hours/week). Smoking status, assessed in 2005, was categorized into the following three classes: never, former, or current smoker. Educational level (< high school diploma, high school to 2-level university, 3- to 4-level university, ≥ 5-level university) was collected in 1992 and marital status (married or not) was collected in 1990. Having farmer parents was collected in 2007 [31].

Body Mass Index (BMI), based on self-reported current weight and height and expressed in kg/m^2^, was calculated in 2005 with the “last observation carried forward” method to take care of missing data, and analysed as a continuous variable or three categories: < 20 [low weight, 20–25 (normal weight) and ⩾25 kg/m^2^ (overweight/obesity) using the WHO cut-off for overweight/obesity [32].

### Statistical analysis

Since the asthma symptom score is a count outcome ranging from 0 to 5, with an excess of zero counts, the strength of the association between the average of the AHEI-2010 score (quintiles) and the asthma symptom score (count) was estimated by using negative binomial regression [33] expressed as mean score ratios (MSRs) and 95% confidence intervals (95% CI). Change in the asthma score is reported for an increase/decrease of 1 category (tertile or quintile) in the AHEI-2010 diet score. Our main model was adjusted for age, energy intake, leisure-time physical activity, smoking status, education level, marital status, and having farmer parents. To account for the study case–control design, models were further adjusted for ever asthma; we also performed a stratified analysis on asthma status, and tested the interaction between quintiles of AHEI-2010 and ever asthma.

Among participants with asthma, associations between tertiles of the AHEI-2010 with uncontrolled asthma (binary outcome) and multimorbidity-related medications profiles (multicategorical outcome) were evaluated by binary logistic and multinomial logistic regressions adjusted for age, energy intake, leisure-time physical activity, smoking status, education level, marital status, and having farmer parents. To handle missing data on ACT (*n* = 729), multiple imputation was performed, as previously reported [34]. The PROC MI SAS procedure was used to perform 20 imputations. We conducted several sensitivity analyses for ACT: (1) using non-imputed data, (2) considering asthma control in three categories (eTable 2), and (3) further adjusting for multimorbidity-related medication profiles.

As obesity is a major risk factor for asthma [35] and healthy diet directly affects obesity [36], it can be hypothesized that obesity acts as a mediator rather than a confounder in the diet-asthma association [37]; therefore, models were not adjusted for BMI. To evaluate effect modification by BMI in the diet-asthma association, we performed sensitivity analyses stratified on BMI and tested the statistical significance of the interaction term. In addition, we used generalized structural equation modeling (SEM), an exploratory approach that enables to test the existence of the hypothesized relationships (i.e. “paths”) between diet, BMI, and the asthma symptom score (eFigure 1) and between diet, BMI, and asthma control (eFigure 2), while controlling for potential confounders [38], using the gsem command in STATA. The association of the BMI with the AHEI-2010 was obtained by linear regression model and measured by the difference in the expected BMI (*β*) according to quintiles or tertiles of the AHEI-2010; associations between BMI and the AHEI-2010 with the asthma symptom score were obtained by negative binomial regression models, and with asthma control through logistic regression models. As obesity is one of the factors included in the LCA to derive the multimorbidity-related medication profiles, we did not stratify on BMI nor apply SEM for this outcome.

To account for potential residual confounding by smoking (never smokers, ex-smokers, current smokers), we also conducted stratified analyses on the smoking status, and tested the statistical significance of interaction term.

The test for trend across AHEI-2010 categories was calculated using quintile or tertile median values.

All tests were two-sided and *p* values < 0.05 were considered statistically significant. Analyses were conducted using SAS version 9.4 (SAS Institute, Cary, NC, USA) or STATA version 14.

## Results

### Participant characteristics

Participant characteristics are shown in Table 1 according to quintiles of the AHEI-2010. Women were aged 63 years on average. Women in the highest quintile of the AHEI-2010 (i.e., healthier diet) were older than those in the lowest quintile (i.e., unhealthier diet). After adjustment for age, women in the highest quintile of the AHEI-2010 consumed fewer calories, were more physically active, less likely to be current smokers, had more often farmer parents, and were less often overweight or obese, as compared to women in the lowest quintile of the AHEI-2010.

**Table 1:** Baseline characteristics of women according to quintiles of the AHEI-2010 diet score (*n* = 12,991)

	AHEI-2010 diet score						
	Quintile 1	Quintile 2	Quintile 3	Quintile 4	Quintile 5	*P* for trend	*P* for trend^a^
AHEI-2010 diet score, min–max	13.0–40.4	40.5–45.3	45.4–49.5	49.6–54.3	54.4–77.0		
AHEI-2010 diet score	35.9 (3.8)	43.1(1.4)	47.4 (1.2)	51.8 (1.3)	58.8 (3.7)		
*Component score of AHEI-2010*							
Vegetables, servings/d	5.4 (2.7)	6.2 (2.6)	6.6 (2.5)	6.9 (2.6)	7.4 (2.5)	** < 0.0001**	** < 0.0001**
Fruits, servings/d	1.4 (0.8)	1.7 (0.9)	1.9 (1.0)	2.1 (1.0)	2.5 (1.1)	** < 0.0001**	** < 0.0001**
Cereal fibres, g/d	2.2 (2.5)	2.7 (2.9)	3.2 (3.2)	3.8 (3.4)	4.7 (3.7)	** < 0.0001**	** < 0.0001**
Sugar-sweetened drinks and fruit juice, servings/d	0.8 (0.7)	0.6 (0.6)	0.5 (0.6)	0.4 (0.5)	0.3 (0.4)	** < 0.0001**	** < 0.0001**
Nuts and legumes, servings/d	0.5 (0.5)	0.7 (0.6)	0.7 (0.7)	0.8 (0.7)	1.0 (0.8)	** < 0.0001**	** < 0.0001**
Red and processed meat, servings/d	1.4 (0.6)	1.2 (0.6)	1.1 (0.5)	1.0 (0.5)	0.9 (0.5)	** < 0.0001**	** < 0.0001**
Long-chain n-3 fatty acids, mg/d	38.8 (24.3)	41.9 (27.0)	43.4 (28.9)	45.2 (30.0)	50.3 (36.3)	** < 0.0001**	** < 0.0001**
PUFA, % of energy	5.1 (1.3)	5.5 (1.3)	5.9 (1.4)	6.3 (1.5)	6.9 (1.7)	** < 0.0001**	** < 0.0001**
Sodium, mg/d	2,971 (841)	2,816 (795)	2,729 (763)	2,605 (719)	2,460 (679)	** < 0.0001**	** < 0.0001**
Alcohol, drinks/d	2.1 (2.2)	1.6 (1.7)	1.4 (1.4)	1.2 (1.1)	1.0 (0.8)	** < 0.0001**	** < 0.0001**
Age (years)	63.1 (6.2)	63.1 (6.1)	63.4 (6.2)	63.6 (6.2)	63.4 (5.9)	**0.01**	
Energy intake (kcal/d)	2,413 (565)	2,345 (549)	2,274 (525)	2,206 (494)	2,166 (480)	** < 0.0001**	** < 0.0001**
Leisure-time physical activity (METs/week)	58.8 (50.7)	61.1 (50.4)	61.9 (52.1)	60.5 (48.1)	63.3 (49.2)	**0.03**	**0.01**
*Smoking status*						** < 0.0001**	** < 0.0001**
Never smoker	1205 (46.6)	1291 (49.7)	1237 (47.8)	1269 (49.3)	1324 (50.0)		
Occasional ex-smoker	276 (10.7)	299 (11.5)	311 (12.0)	307 (11.9)	284 (10.7)		
Regular ex-smoker	647 (25.0)	659 (25.4)	722 (27.9)	749 (29.1)	784 (29.6)		
Occasional current smoker	35 (1.4)	32 (1.2)	30 (1.2)	24 (1.0)	34 (1.3)		
Regular current smoker	172 (6.7)	147 (5.7)	123 (4.8)	83 (3.2)	105 (3.9)		
Missing	249 (9.6)	170 (6.5)	162 (6.3)	142 (5.5)	119 (4.5)		
*Educational level*						0.14	0.07
< high school diploma	256 (9.9)	251 (9.7)	239 (9.3)	445 (9.4)	498 (8.0)		
High school to 2-level university	1257 (48.7)	1286 (49.5)	1301 (50.3)	1343 (52.2)	1340 (50.5)		
3- to 4-level university	504 (19.5)	471 (18.1)	480 (18.6)	477 (18.5)	517 (19.5)		
≥ 5-level university	481 (18.6)	522 (20.1)	483 (18.7)	445 (17.3)	498 (18.8)		
Missing	86 (3.3)	68 (2.6)	82 (3.1)	66 (2.6)	84 (3.2)		
*Marital status*						0.19	0.65
No	438 (17.0)	424 (16.3)	379 (14.7)	400 (15.5)	428 (16.2)		
Yes	2044 (79)	2098 (80.8)	2112 (81.7)	2098 (81.5)	2123 (80.1)		
Missing	102 (4.0)	76 (2.9)	94 (3.6)	76 (3.0)	99 (3.7)		
*Having farmer parents*						** < 0.0001**	** < 0.0001**
No	2304 (89.1)	2253 (86.7)	2202 (85.2)	2228 (86.6)	2249 (84.9)		
Yes	206 (8.0)	269 (10.4)	298 (11.5)	278 (10.8)	332 (12.5)		
Missing	74 (2.9)	76 (2.9)	85 (3.3)	68 (2.6)	69 (2.6)		
*BMI (kg/m*^*2*^*)*						** < 0.0001**	** < 0.0001**
< 20	288 (11.1)	309 (11.9)	312 (12.1)	314 (12.2)	362 (13.6)		
20–24.9	1354 (52.4)	1408 (54.2)	1435 (55.5)	1468 (57.0)	1570 (59.3)		
25–29.9	716 (27.7)	673 (25.9)	658 (25.5)	628 (24.4)	594 (22.4)		
≥ 30	226 (8.8)	208 (8.0)	180 (6.9)	164 (6.4)	124 (4.7)		
BMI (kg/m^2^)	24.3 (4.1)	24.1 (4.0)	24.0 (4.0)	23.8 (3.7)	23.4 (3.5)	** < 0.0001**	** < 0.0001**
*Asthma symptom score*^a^						** < 0.0001**	** < 0.0001**
0	1502 (58.1)	1540 (59.3)	1560 (60.3)	1581 (61.4)	1704 (64.3)		
1	680 (26.3)	733 (28.2)	693 (26.8)	661 (25.7)	678 (25.6)		
2	178 (6.9)	170 (6.5)	185 (7.2)	180 (7.0)	129 (4.9)		
≥ 3	224 (8.7)	155 (6.0)	147 (5.7)	152 (5.9)	139 (5.2)		
*Asthma control test*^c^						0.20	0.13
> 19	490 (75.3)		501 (79.2)		440 (76.7)		
≤ 19	161 (24.7)		132 (20.8)		134 (23.3)		
*Multimorbidity-related medication profiles*^c^						0.07	**0.03**
“Few multimorbidity-related medications”	510 (43.3)		508 (44.0)		532 (46.6)		
“Predominantly allergic multimorbidity-related medications”	391 (33.2)		380 (32.9)		376 (32.9)		
“Predominantly metabolic multimorbidity-related medications”	276 (23.5)		267 (23.1)		234 (20.5)		

*P* < 0.05 values are presented in bold

Data are presented as *n* (%) or mean (SD) unless otherwise stated. *P* for trend were calculated using the quintile median values

^a^Age-adjusted models

^b^Asthma symptom score: number of respiratory symptoms during the past 12 months: 1) breathless while wheezing; 2) woken up with chest tightness; 3) attack of shortness of breath at rest; 4) attack of shortness of breath after exercise; and 5) woken by attack of shortness of breath. Each item is scored from zero to one and the total asthma symptom score ranges from zero to five [21]

^c^For ACT and multimorbidity-related medications profiles, distributions are given according to tertiles of the AHEI-2010 diet core (instead of quintiles)

ACT: asthma control test, based on five questions in the last 4 weeks on: 1. activity limitations (does asthma keep you from getting as much done at work, school or at home, “some of the time” to “all of the time”); 2. shortness of breath (“three to six times per week” to “more than once daily”); 3. woken up by asthma symptoms at night (“once per week” to “every night”); 4. use of a β-agonist inhaler (“two times per week” to “three or more times daily”); and 5. self-rated asthma control (“somewhat controlled” to “not controlled at all”). Each item is scored from one to five and the total ACT score ranges from five to 25. Asthma control was classified into two categories based on ACT score (> 19: controlled vs. ⩽19: uncontrolled) [22]

Multimorbidity-related medications profiles: using a clustering method, we previously identified three multimedication profiles among women with asthma: a “Few multimorbidity-related medications” profile, a “Predominantly allergic multimorbidity-related medications” profile, and a “Predominantly metabolic multimorbidity-related medications” profile [23]

### Association between the AHEI-2010 and the asthma symptom score

Thirty-nine percent of women reported at least one asthma symptom. After adjustment for age (Table 2), women with higher AHEI-2010 scores had lower asthma symptom scores with MSRs (95% CI) of 0.91 (0.83–0.99) for quintile 2 (Q2), 0.85 (0.78–0.93) for quintile 3 (Q3), 0.86 (0.79–0.94) for quintile 4 (Q4), and 0.77 (0.71–0.85) for quintile 5 (Q5) as compared with the first quintile (*p* trend < 0.001). After adjustment for potential confounders (model 2), the association remained of similar magnitude. Further adjustment for ever asthma led to a similar result (model 3).

**Table 2:** Association between the AHEI-2010 diet score (quintiles) and the asthma symptom score (*n* = 12,991)

			Age-adjusted model 1	Multivariable-adjusted model 2^a^	Multivariable-adjusted model 3^b^
	*N*	AHEI-2010, mean (sd)	MSR (95% CI)	MSR (95% CI)	MSR (95% CI)
*AHEI-2010 diet score*	12,991				
Quintile 1	2,584	35.9 (3.8)	1.00 (ref)	1.00 (ref)	1.00 (ref)
Quintile 2	2,598	43.1 (1.4)	**0.91 (0.83–0.99)**	**0.92 (0.84–1.00)**	**0.92 (0.85–1.00)**
Quintile 3	2,585	47.4 (1.2)	**0.85 (0.78–0.93)**	**0.85 (0.78–0.93)**	**0.87 (0.80–0.95)**
Quintile 4	2,574	51.8 (1.3)	**0.86 (0.79–0.94)**	**0.87 (0.79–0.95)**	**0.90 (0.82–0.98)**
Quintile 5	2,650	58.8 (3.7)	**0.77 (0.71–0.84)**	**0.80 (0.73–0.88)**	**0.82 (0.75–0.90)**
P for trend			** < 0.0001**	** < 0.0001**	** < 0.0001**

*P* < 0.05 values are presented in bold

*MSR* mean score ratio

*P* for trend were calculated using the quintile median values

^a^Multivariable-adjusted model 2 includes age, energy intake, physical activity, smoking, educational level, marital status and having farmer parents

^b^Multivariable-adjusted model 3 includes model 2 variables (see above) plus ever asthma

**Fig. 2:**
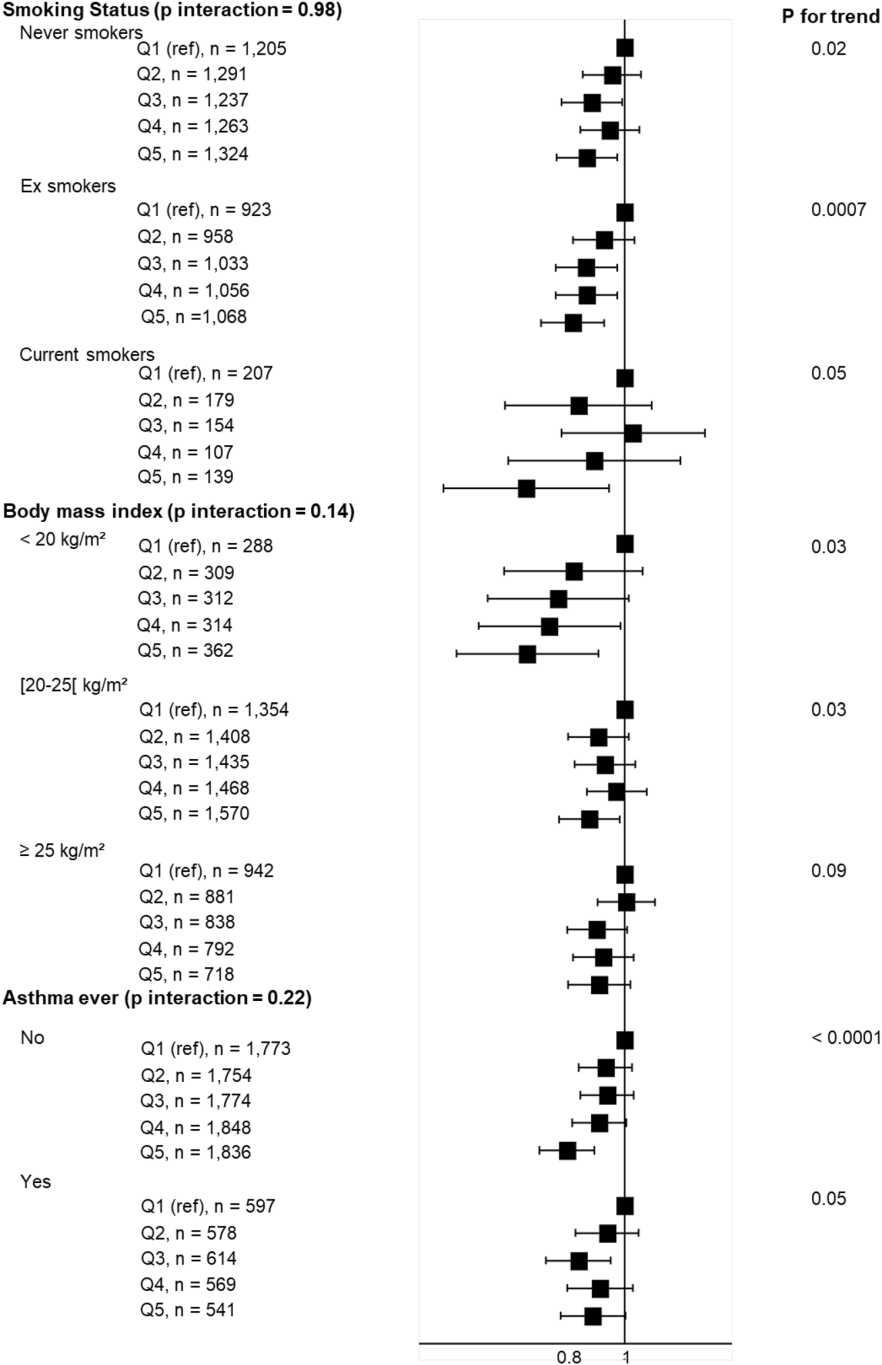
Associations between quintiles of the AHEI-2010 diet score and the asthma symptom score, stratified according to smoking status, body mass index (BMI) and ever asthma. Models were adjusted for age, energy intake, physical activity, smoking (excepted in models stratified by smoking status), educational level, marital status, and having farmer parents. The first quintile (Q1) serves as reference. Asthma symptom score ranges from 0 to 5

In analyses stratified on BMI, MSRs remained below one in each category, and the interaction term between the AHEI-2010 and BMI was not statistically significant (Fig. 2) (*p* for interaction = 0.14). Using SEM, we still reported a negative association between the AHEI-2010 and the asthma symptom score, as well a significant negative dose–response relationship between the AHEI-2010 and BMI and a significant positive association between BMI and the asthma symptom score (eFigure 1).

In analyses stratified on smoking status, associations remained statistically significant and of similar magnitude in each category (Fig. 2) (*p* for interaction = 0.98). Finally, when analyses were stratified according to the asthma status, similar associations were reported between the AHEI-2010 and the asthma symptom score among women with or without self-reported ever asthma (*p* for interaction = 0.22).

### Association between AHEI-2010 and uncontrolled asthma

Among women with current asthma, 23% had uncontrolled asthma. After adjustment for age (Table 3), only the second tertile of the AHEI-2010 was associated with a lower likelihood of uncontrolled asthma: OR (95% CI) was 0.73 (0.56–0.94) for tertile 2, and 0.82 (0.64–1.06) for tertile 3, as compared with tertile 1, *p* for trend = 0.19. After adjustment for potential confounders (model 2), and further adjustment for multimorbidity-related medication profiles (model 3), associations became weaker. Similar findings were reported when using the non-imputed dataset (*n* = 1,858; Table 3) or the ACT categorised into three categories (etable 2).

**Table 3:** Association between the AHEI-2010 diet score (tertiles) and uncontrolled asthma

			Age-adjusted model 1	Multivariable-adjusted model 2^b^	Multivariable-adjusted model 3^c^
	*n*	AHEI-2010, mean (sd)	OR (95% CI)	OR (95% CI)	OR (95% CI)
*Uncontrolled asthma (after imputation)*^a^	2587				
AHEI-2010 tertile 1	895	38.3 (4.4)	1.00 (ref)	1.00 (ref)	1.00 (ref)
AHEI-2010 tertile 2	884	47.4 (2.0)	**0.73 (0.56–0.94)**	**0.72 (0.55–0.94)**	**0.73 (0.55–0.98)**
AHEI-2010 tertile 3	808	56.1 (4.3)	0.82 (0.64–1.06)	0.84 (0.65–1.10)	0.86 (0.65–1.12)
*P* for trend			0.11	0.19	0.24
*Uncontrolled asthma (before imputation)*	1858				
AHEI-2010 tertile 1	651	38.2 (4.5)	1.00 (ref)	1.00 (ref)	1.00 (ref)
AHEI-2010 tertile 2	633	47.4 (2.0)	**0.76 (0.57–1.00)**	**0.75 (0.57–1.00)**	**0.76 (0.57–1.00)**
AHEI-2010 tertile 3	574	55.9 (4.2)	0.87 (0.66–1.14)	0.89 (0.67–1.18)	0.92 (0.69–1.2)
*P* for trend			0.32	0.39	0.51

*P* < 0.05 values are presented in bold

*P* for trend were calculated using the tertile median values

^a^Analysis after using multiple imputation to estimate ACT missing values, as in previous analyses in this population [40]. See online supplement for details

^b^Multivariable-adjusted model 2 includes age, energy intake, physical activity, smoking, educational level, marital status and having farmer parents

^c^Multivariable-adjusted model 3 includes model 2 variables (see above) plus multimorbidity-related medication profiles

**Fig. 3:**
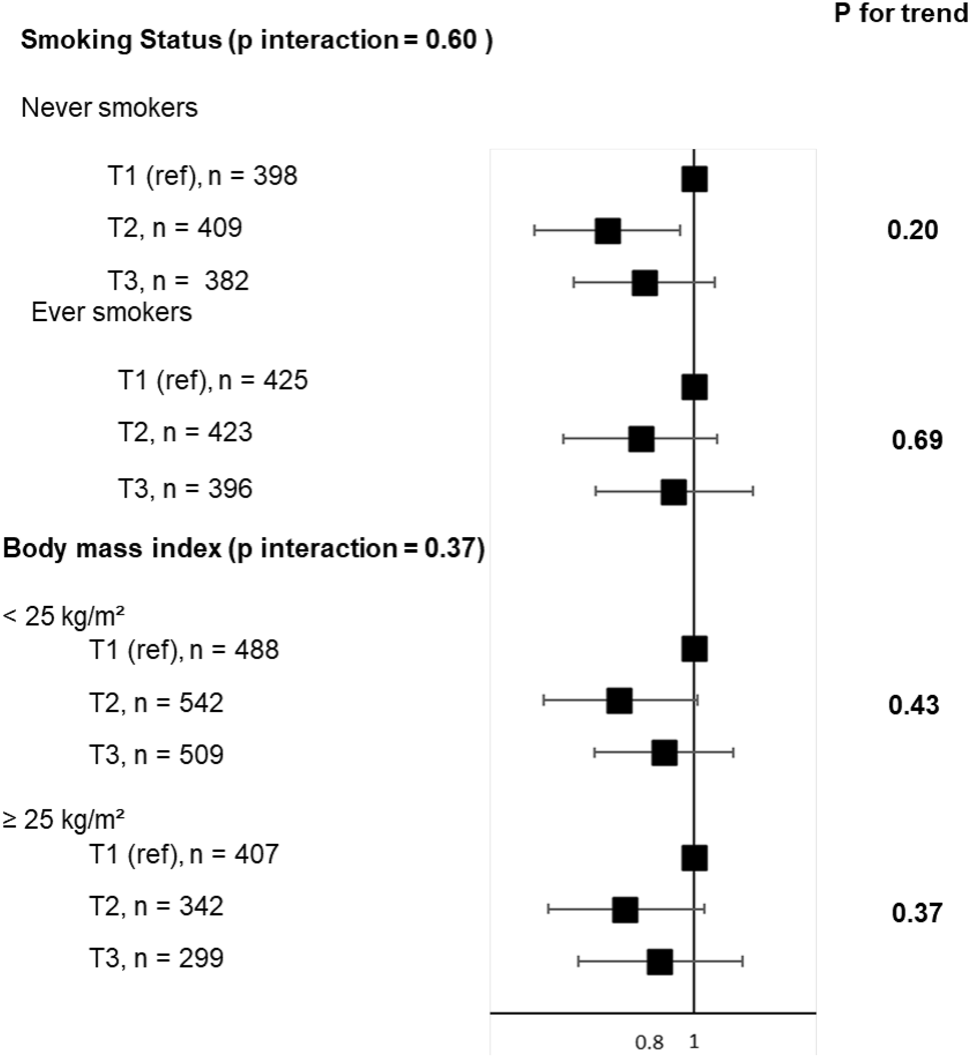
Associations between tertiles of the AHEI-2010 diet score and uncontrolled asthma, stratified according to smoking status and BMI. Models were adjusted for age, energy intake, physical activity, smoking (excepted in models stratified by smoking status), educational level, marital status and having farmer parents. The first tertile (T1) serves as reference

In analyses stratified on BMI, similar associations were reported, and the interaction term between the AHEI-2010 and BMI was not statistically significant (Fig. 3) (*p* for interaction = 0.34). Using SEM, we still reported no significant association between the AHEI-2010 and uncontrolled asthma, as well a significant negative relationship between the AHEI-2010 and BMI, and a significant positive association between BMI and uncontrolled asthma (eFigure 2).

After stratification for smoking, associations between the AHEI-2010 and uncontrolled asthma remained of similar magnitude in each category with no statistical significance, except for AHEI-tertile 2 in never smokers (Fig. 3) (*p* for interaction = 0.64).

### Association between AHEI-2010 and multimorbidity-related medication profiles

Among women with ever asthma, 44.7% belonged to the “Few multimorbidity-related medications”, 33% belonged to the “predominantly metabolic multimorbidity-related medications” profile, and 22.3% to the “predominantly allergic multimorbidity-related medications” profile. After adjustment for age (Table 4), women with higher AHEI-2010 score were at a lower risk to belong to the “Predominantly metabolic multimorbidity-related medications” profile compared to the “Few multimorbidity-related medications" profile: ORs (95% CI) were 0.94 (0.76–1.17) for tertile 2 and 0.75 (0.60–0.93) for tertile 3 vs. tertile 1 (*p* trend = 0.01). Results remained similar after further adjustment for potential confounders. By contrast, the AHEI-2010 was not associated with the “predominantly allergic multimorbidity-related medications” profile.

**Table 4:** Association between the AHEI-2010 diet score (tertiles) and multimorbidity-related medication profiles (*n* = 3474)

	Multimorbidity-related medication profiles								
	“Few multimorbidity-related medications” profile			“Predominantly allergic multimorbidity-related medications” profile			“Predominantly metabolic multimorbidity-related medications” profile		
	*n*	AHEI-2010, mean (sd)	OR (95% CI)	*n*	AHEI-2010, m (sd)	OR (95% CI)	*n*	AHEI-2010, *m* (sd)	OR (95% CI)
*Age-adjusted model 1*	1550			1147			777		
AHEI-2010 tertile 1	510	38.7 (4.1)	1.00 (ref)	391	38.1 (4.5)	1.00 (ref)	276	38.1 (4.3)	1.00 (ref)
AHEI-2010 tertile 2	508	47.3 (2.0)	1.00 (ref)	380	47.5 (2.1)	0.97 (0.80–1.17)	267	47.3 (2.0)	0.94 (0.76–1.17)
AHEI-2010 tertile 3	532	56.4 (4.4)	1.00 (ref)	376	56.2 (4.3)	0.91 (0.75–1.10)	234	55.5 (4.2)	**0.75 (0.60–0.93)**
*P* for trend						0.32			**0.01**
*Multivariable-adjusted model 2*^a^	1550			1147			777		
AHEI-2010 tertile 1	510	38.7 (4.1)	1.00 (ref)	391	38.1 (4.5)	1.00 (ref)	276	38.1 (4.3)	1.00 (ref)
AHEI-2010 tertile 2	508	47.3 (2.0)	1.00 (ref)	380	47.5 (2.1)	0.99 (0.81–1.20)	267	47.3 (2.0)	0.96 (0.77–1.21)
AHEI-2010 tertile 3	532	56.4 (4.4)	1.00 (ref)	376	56.2 (4.3)	0.96 (0.79–1.17)	234	55.5 (4.2)	**0.80 (0.63–1.00)**
*P* for trend						0.64			**0.05**

*P* < 0.05 values are presented in bold

*P* for trend were calculated using the tertile median values

^a^ + Multivariable-adjusted model 2 includes age, energy intake, physical activity, smoking, educational level, marital status and having farmer parents

**Fig. 4:**
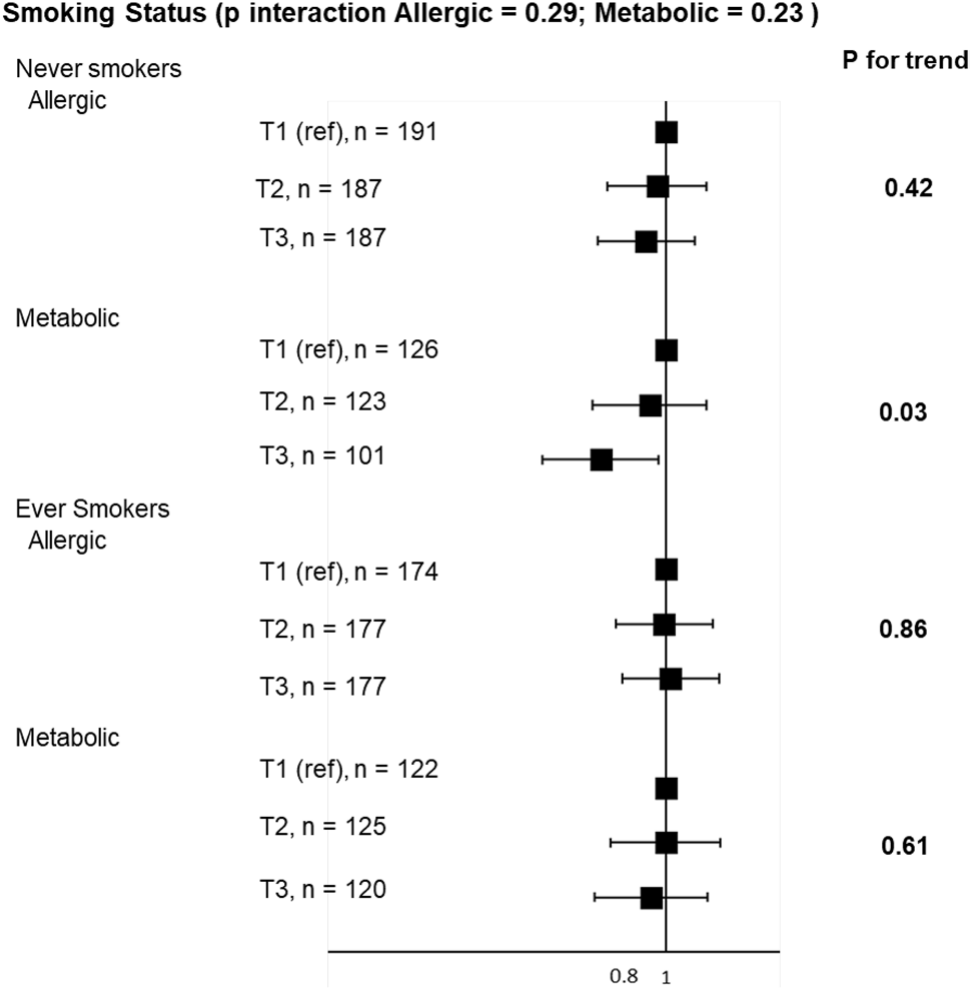
Associations between tertiles of the AHEI-2010 diet score and multimorbidity-related medication profiles, stratified according to smoking status. Models were adjusted for age, energy intake, physical activity, educational level, marital status and having farmer parents. The first tertile (T1) serves as reference

After stratification for smoking (Fig. 4), similar associations were reported between the AHEI-2010 and multimorbidity-related medication profiles among never and ever smokers, with no interaction between the AHEI-2010 and the smoking status (*p* = 0.29 for the allergic profile, and 0.23 for the metabolic profile).

## Discussion

In this large study of more than 12,000 elderly women, a stronger adherence to a healthy diet evaluated by the AHEI-2010 was associated with a lower asthma symptom score, and among women with asthma, with disease characteristics associated with better asthma prognosis. These findings strengthen evidence supporting the promotion of a healthy diet to target reduction in asthma respiratory symptoms among elderly women, and confirm the importance of considering diet in the relation between asthma and comorbidities, especially cardiovascular diseases.

To our knowledge, five studies have investigated the association between the AHEI-2010 and asthma outcomes, among young [39] to middle-aged adults [17, 18, 40, 41], and using a dichotomous [39–41] or a continuous [17, 18] definition of asthma, and they reported mixed findings. The three studies that used a dichotomous definition of asthma, namely wheezing (yes/no), incident asthma (yes/no), or at least one out of three current asthma symptoms (yes/no), reported conflicting results. Using the asthma symptom score, two previous studies have investigated its association with the AHEI-2010 and reported that a healthy diet was associated with lower asthma symptoms [17, 18]. Regarding other dietary scores (a priori approach), the most studied are the Mediterranean diet score based on recommended foods or nutrients for disease prevention (such as the AHEI-2010) and the Dietary Inflammatory Index (DII) which relates to pathophysiological processes relevant to asthma (i.e. inflammation). To our knowledge, only one cross-sectional study reported a positive association between the Mediterranean diet and the asthma symptoms score in adults [18]. For the DII, one case–control [42] and two cross-sectional studies [41, 43] investigated associations with asthma in adults, and they all reported that a higher DII was associated with asthma [42] or current asthma [41, 43]. By contrast, using the a posteriori approach to derive data-driven dietary patterns (i.e., statistically derived independently of their relevance to any disease), at least 13 studies have looked at the association between dietary patterns, mostly derived using principal component analysis (PCA), and asthma symptoms or incidence in adults, and they have yielded mixed findings [44–47]. Our current findings are consistent with those based on younger populations and support the likely impact of a healthy diet in the prevention of asthma in elderly women. They extend results obtained from younger population and provide first evidence for the impact of a healthy diet in the prevention of asthma over the life course.

Regarding the impact of an overall healthy diet as a disease modifier, a healthier diet was associated in our study with a lower risk of uncontrolled asthma but the association did not reach statistical significance. Five studies have been published so far, all among middle-aged adults and overall, they reported mixed findings as follows: three studies reported an association between an overall healthy diet and lower uncontrolled asthma [48–50], one reported no association [51], and one reported a borderline significant association in women [18]. Published studies are very heterogeneous in term of tools used to evaluate asthma control as follows: two used the ACT [18, 50] and three used the asthma control questionnaire (ACQ) [48, 49, 51]; in terms of tools used to collect dietary data, three used 24-h dietary records [18, 48, 50], and two used a FFQ [49, 51]; and in terms of tools used to estimate the overall diet, one used the Dietary Approaches to Stop Hypertension (DASH) score [48], one used the DII [50], two used the Mediterranean diet score [49, 51], and one used the AHEI-2010 [18]. All five studies used dietary data collected at the same time as asthma control data or in the year before. In our study, diet was evaluated by two validated semi-quantitative food history questionnaires administrated in 1993 and 2005, whereas asthma control was evaluated in 2011. The assessment of diet does not cover the ACT window of exposure and it is likely that participants may have modified their diet between 1993 and 2011. Anyhow, it has been shown that diet remains globally stable in this specific population, namely elderly women with a high level of education [52], and we used the average diet between 1993 and 2005 which is likely more representative of the usual diet as compared to one assessment only. Although diet might be a modifiable risk factor that could be targeted to help reduce asthma exacerbations and reach a good level of asthma control, its potential role on asthma control remains unclear, particularly among elderly women.

Besides allergic-related morbidities, that are common especially in childhood asthma, it has been more recently reported that mechanisms involved in adult-onset asthma also include several metabolic and inflammatory pathways also related to other chronic conditions such as obesity, the metabolic syndrome, type 2 diabetes mellitus, or CVD [53]. Although the underlying mechanisms remain unknown, it has been suggested that asthma and CVD share common risk factors such as smoking, obesity, or more recently reported, air pollution exposure [54], consistent with common etiological pathways. The AHEI-2010 is based on a comprehensive review to identify foods and nutrients that have been consistently associated with lower risks of CVD, cancer, and type 2 diabetes in clinical and epidemiological investigations [16]. The AHEI-2010 also captures additional information on diet quality that may further decrease the risk of metabolic diseases [16]. Our findings provide further evidence for a major role of an unhealthy diet as a common risk factor between asthma and CVD. A better understanding of the role of a healthy diet could lead to risk management strategies for patients with asthma to reduce their CVD risk.

Several hypotheses and mechanisms have been raised to explain the role of diet in asthma, including oxidative stress and inflammation, and more recently, vitamin D, epigenetic regulation, and imbalance in the gut microbiome [55]. Asthma is a chronic inflammatory disease of the airways, and endogenous reactive oxygen species have been implicated in its pathogenesis [56]. It has been extensively reported that a better quality diet (high in fruit, vegetables, whole grains, and legumes as reflected by a high AHEI-2010) is associated with lower inflammatory biomarkers [57], and more recently with a reduction in short-chain fatty acids [58] (produced by bacteria in the gut during fermentation of fibre from dietary plant matter) known to reduce airway inflammation [59]. In this manner, a dietary intervention based on a fibre-rich healthy diet might be relevant for both primary and secondary prevention of asthma.

Diet, physical activity, and body composition are nutritional factors that are not only closely interrelated (at a given time *t*) but also time-dependent, which makes it difficult to disentangle their separate effects on asthma outcome [60]. Regarding interrelations (at a given time *t*), although obesity can result from multiple factors (e.g., genetic predispositions, certain disease status, or medication use), it is most often a result of unhealthy lifestyle, including excessive dietary energy intakes and insufficient physical activity [61]. It has been shown that individuals with lower overall diet quality have higher risk of obesity [36]. The established evidence that links diet to obesity and that links obesity to asthma [10] and asthma control [62] can illustrate the role of obesity as a potential mediator in the diet–asthma association [60]. In this context, several studies provided results with and, in addition, without adjustment for BMI [63, 64] but it could lead to biased results [65]. Some novel analyzing approaches, such as the counterfactual approach, provide a new tool to face the above issues in mediation analysis [66]. To our knowledge, only two studies were conducted in the context of nutritional factors and asthma, suggesting that BMI partly mediates the association between high cured meat intake and worsening asthma symptoms over time [67], but does not mediate the association between overall diet quality—assessed using the AHEI-2010—and improved asthma symptoms [17]. From a longitudinal perspective, interrelations between nutritional factors and asthma are also time-dependent. Indeed, in addition to the potential role of each nutritional factor at a given time *t* on asthma at a time *t* + 1, asthma at time *t* − 1 may have modified nutritional factors at time *t* (e.g., asthma can lead to a decrease in physical activity), and each nutritional factor at time *t* − 1 may have modified another nutritional factor at time *t* (e.g., overweight/obesity can lead to modifying dietary and/or physical activity behaviours). To our knowledge, only one study has been conducted in the context of the time-dependent associations between physical activity, BMI and asthma (also using data from the E3N study) and suggested an independent causal deleterious effect of overweight and obesity on current asthma, but no independent causal effect of physical activity on current asthma [30]. Although longitudinal data are warranted to fully address these issues, our models stratified according to BMI yielded to similar associations between diet and asthma outcomes, and estimates obtained from SEM and those obtained from standard models are very close, showing that BMI is unlikely a major modifier or mediator in the association between diet with asthma outcomes.

Our study has several strengths and limitations. First, our analysis was cross-sectional. However, our findings are based on a large sample size, which allows accounting for several potential confounders and performing stratified analyses to address the robustness of the findings. Secondly, diet was evaluated in 1993 and 2005, and asthma in 2011. Although participants may have modified their diet between 1993 and 2011, it has been shown that the diet remains globally stable in this specific population of elderly women with a high level of education [52]. Although a substantial proportion of women did not complete the ACT (28%), analyses with and without imputed data yielded similar results. Besides, associations were consistent in several sub-populations and after adjustment for many potential confounders. In addition, we used validated tools to estimate asthma symptoms [24, 25] and asthma control [26] as well as the diet [68]. Secondly, we acknowledge that the main source of disease misclassification among this population of elderly women is probably misdiagnosis of COPD, and that potential overlap between asthma and COPD may have contributed to the association between the AHEI-2010 and asthma. However, we used the asthma symptom score that measures specific symptoms of asthma (and not of COPD) rather than a dichotomous definition of asthma which is more likely to include COPD patients. Moreover, similar associations were observed whatever the smoking status, especially among never smokers who are less likely to suffer from COPD. We also acknowledge that the association between a healthy diet and the “Predominantly metabolic multimorbidity-related medications” profile might be due in part, to obesity. Indeed, obesity (e.g., BMI ≥ 30 kg/m^2^) was included in the LCA to identify asthma groups, and whereas the “Few multimorbidity-related medications” profile and the “Predominantly allergic multimorbidity-related medications” include respectively 3.4% and 1.5% of obese women, the “Predominantly metabolic multimorbidity-related medications” profile includes 34% of obese women. Finally, the relative homogeneity of the studied population (e.g., elderly women with mostly high educational levels) actually helps with causal inferences about the relation between healthy diet and asthma outcomes because the comparability of the high and low dietary score groups will be higher than in a more heterogeneous populations (i.e., less potential for residual confounding).

In this elderly female population, we observed that a healthier dietary intake was associated with lower asthma symptoms, and among women with asthma, lower risk to belong to a metabolic multimorbidity-related medication profile. Overall, our findings show that a healthy diet may play an important role in the prevention and management of asthma over the life course. There is a need for longitudinal studies and RCTs to help understand better the role of the AHEI-2010 diet for the primary and secondary prevention of asthma among elderly. More studies are also warranted to better understand the role of a healthy diet as a common risk factor for patients with asthma to better reduce their CVD risk, which could lead to risk management strategies among those patients. Lastly, as the investigation of nutritional factors as a whole (diet, physical activity, obesity) is highly relevant in the etiology of asthma and its control, both in terms of understanding the underlying mechanisms and in terms of guiding efficient multidimensional public health interventions, there is a crucial need for further prospective studies to address this issue.

## Supplementary Information

Below is the link to the electronic supplementary material.

Supplementary file1 (DOCX 62 KB)

Supplementary file2 (DOCX 35 KB)

## Data Availability

Requests for access to data, statistical code, questionnaires, and technical processes may be made by contacting the corresponding author at marie-christine.boutron@gustaveroussy.fr.

## Declarations

### Code availability

Statistical Analysis Software (SAS) were used.
