# Supplementary material for: Healthy diet associated with better asthma outcomes in elderly women of the French Asthma-E3N study
Source: Eur J Nutr. 2022 Feb 27;61(5):2555–69. doi: 10.1007/s00394-022-02815-0 (PMC9279211; doi:10.1007/s00394-022-02815-0)
Supplement: Supplementary file 1 — Supplementary file1 (DOCX 62 KB) [file 394_2022_2815_MOESM1_ESM.docx]

**Supplementary Online Content**

**Healthy diet and asthma outcomes among elderly women from the French Asthma-E3N study**

Wassila Ait-hadad, Annabelle Bédard, Sébastien Chanoine, Orianne Dumas, Nasser Laouali, Nicole Le Moual, Bénédicte Leynaert, Conor Macdonald, Valérie Siroux, Marie-Christine Boutron-Ruault, Raphaëlle Varraso

**eTable 1.** Baseline characteristics of included and excluded women

**eTable 2.** Association between the AHEI-2010 diet score (tertiles) and uncontrolled asthma in 3 categories

**eFigure 1.** Associations of AHEI-2010, body mass index and the asthma symptom score using structural equation modeling (SEM)

**eFigure 2.** Associations of AHEI-2010, body mass index and uncontrolled asthma using structural equation modeling (SEM)

**eTable 1.** Baseline characteristics of included and excluded women

|  | Included (n=12,991) | Excluded dietary data^a^ (n=3,708) | P | Age-adjusted p | Excluded asthma data  (n=2,705) | P | Age-adjusted p |
| --- | --- | --- | --- | --- | --- | --- | --- |
| AHEI-2010, m (sd) | 47.5 (8.2) | / | / | / | 47.2 (8.1) | 0.08 | 0.14 |
| Component score of AHEI-2010, m (sd) |  |  |  |  |  |  |  |
| Vegetables, servings/d | 6.5 (2.7) | / | / | / | 6.6 (2.8) | 0.11 | 0.52 |
| Fruits, servings/d | 1.9 (1.0) | / | / | / | 1.9 (1.1) | 0.82 | 0.55 |
| Cereal fibers, g/d | 3.3 (3.2) | / | / | / | 3.3 (3.2) | 0.50 | 0.59 |
| Sugar-sweetened drinks and fruit juice, servings/d | 0.5 (0.6) | / | / | / | 0.5 (0.6) | 0.94 | 0.76 |
| Nuts and legumes, servings/d | 0.8 (0.7) | / | / | / | 0.8 (0.7) | 0.95 | 0.16 |
| Red and processed meat, servings/d | 1.1 (0.6) | / | / | / | 1.2 (0.6) | **0.02** | **0.0004** |
| Long-chain n-3 fatty acids, mg/d | 44.0 (29.9) | / | / | / | 44.9 (31.5) | 0.17 | 0.22 |
| PUFA, % of energy | 5.9 (1.6) | / | / | / | 5.9 (1.6) | 0.33 | 0.40 |
| Sodium, mg/d | 2,715 (781) | / | / | / | 2,745 (784) | 0.07 | **0.008** |
| Alcohol, drinks/d | 1.5 (1.6) | / | / | / | 1.5 (1.6) | 0.58 | 0.27 |
| Age (years), m (sd) | 63.3 (6.1) | 65.1 (6.7) | **<0.0001** | / | 65.1 (6.5) | **<0.0001** | / |
| Energy intake(kcal/d), m (sd) | 2,279 (530) | / | / | / | 2,290 (534) | 0.33 | **0.02** |
| Leisure-time physical activity, MET/week | 61.2 (50.1) | 58.5 (50.7) | **0.01** | 0.69 | 58.1 (50.8) | **0.006** | **0.02** |
| Smoking status, n (%) |  |  | **0.0001** | 0.09 |  | 0.35 | **0.04** |
| Never smoker | 6,326 (48.7) | 1,516 (40.9) |  |  | 1,279 (47.3) |  |  |
| Occasional ex-smoker | 1,477 (11.4) | 299 (8.1) |  |  | 297 (11.0) |  |  |
| Regular ex-smoker | 3,561 (27.4) | 916 (24.7) |  |  | 727 (26.9) |  |  |
| Occasional current smoker | 155 (1.2) | 45 (1.2) |  |  | 26 (1.0) |  |  |
| Regular current smoker | 630 (4.9) | 201 (5.4) |  |  | 152(5.6) |  |  |
| Missing | 842 (6.5) | 731(19.7) |  |  | 224 (8.3) |  |  |
| Educational level, n (%) |  |  | **<0.0001** | **<0.0001** |  | **<0.0001** | **<0.0001** |
| < high school diploma | 1,200 (9.2) | 686 (18.5) |  |  | 385 (14.2) |  |  |
| High school to 2-level university | 6527 (50.2) | 1702 (45.9) |  |  | 1296 (47.9) |  |  |
| 3- to 4-level university | 2449 (18.9) | 595 (16.1) |  |  | 451 (16.7) |  |  |
| ≥ 5-level university | 2429 (18.7) | 557 (15.1) |  |  | 449 (16.6) |  |  |
| Missing | 386 (3.0) | 168 (4.5) |  |  | 124 (4.6) |  |  |
| Marital status, n (%) |  |  | **<0.0001** | **0.005** |  | **0.02** | 0.08 |
| No | 2,069 (15.9) | 684 (18.5) |  |  | 472 (17.5) |  |  |
| Yes | 10,475 (80.6) | 2,798 (75.5) |  |  | 2,097 (77.5) |  |  |
| Missing | 447(3.4) | 226 (6.1) |  |  | 136 (5.0) |  |  |
| Having farmer parents, n (%) |  |  | 0.15 | 0.39 |  | 0.71 | 0.80 |
| No | 11,236 (86.5) | 2,907 (78.4) |  |  | 2,325 (86.0) |  |  |
| Yes | 1,383 (10.7) | 391 (10.5) |  |  | 279 (10.3) |  |  |
| Missing | 372 (2.9) | 410 (11.1) |  |  | 101 (3.7) |  |  |
| BMI (kg/m²), n (%) |  |  | **<0.0001** | **<0.0001** |  | **<0.0001** | **<0.0001** |
| < 20 | 1,633 (12.6) | 92 (2.5) |  |  | 311 (11.5) |  |  |
| [20-25[ kg/m² | 7,270 (56.0) | 277 (7.5) |  |  | 1,444 (53.4) |  |  |
| [25-30[ kg/m² | 3,194 (24.6) | 182 (4.9) |  |  | 686 (25.4) |  |  |
| ≥ 30 kg/m² | 894 (6.9) | 73 (2.0) |  |  | 264 (9.8) |  |  |
| Missing | 0 (0) | 3,084 (83.2) |  |  | 0 (0) |  |  |
| Asthma symptom score, n (%) |  |  | **<0.0001** | **<0.0001** |  | / | / |
| 0 | 7,887 (50.3) | 1,673 (45.1) |  |  | / |  |  |
| 1 | 3,445 (22.0) | 716 (19.3) |  |  | / |  |  |
| 2 | 842 (5.4) | 205 (5.5) |  |  | / |  |  |
| 3 | 407 (2.6) | 115 (3.1) |  |  | / |  |  |
| 4 | 228 (1.5) | 78 (2.1) |  |  | / |  |  |
| 5 | 182 (1.2) | 57 (1.5) |  |  | / |  |  |
| Missing | 2,705 (17.2) | 864 (23.3) |  |  |  |  |  |
| **Asthma control test, n (%)** | *n = 2,587* | *680* | **0.001** | **0.01** |  | / | / |
| >19 | 1,431 (77.0) | 334 (69.9) |  |  | / |  |  |
| ≤19 | 427 (23.0) | 144 (30.1) |  |  | / |  |  |
| Missing | 729 (28.2) | 202 (29.7) |  |  |  |  |  |
| **Multimorbidity-related medication profiles** | *n = 3,727* | *937* | **0.0002** | **0.003** |  | / | / |
| Few multimorbidity | 1550 (44.6) | 335 (39.2) |  |  | / |  |  |
| Allergic multimorbidity | 1147 (33.0) | 272 (31.9) |  |  | / |  |  |
| Metabolic multimorbidity | 777 (22.4) | 247 (28.9) |  |  | / |  |  |
| Missing | 253 (6.8) | 83 (8.9) |  |  |  |  |  |

^a^ Excluded women because they did not complete the food questionnaire in 1993 or in 2005, or had an implausibly high or low total energy intake in 1993 or in 2005. ^b^ Excluded women because they did not answer to the asthma symptom score questions.

**eTable 2.** Association between the AHEI-2010 diet score (tertiles) and uncontrolled asthma in 3 categories

|  | **Asthma control test** | | | | | | | | |
| --- | --- | --- | --- | --- | --- | --- | --- | --- | --- |
|  | ACT ≥ 25 (controlled) | | | 20 ≤ ACT ≤ 24 (partly controlled) | | | ACT ≤ 19 (poorly controlled) | | |
|  | n | AHEI-2010, m (sd) | OR (95% CI) | n | AHEI-2010, m (sd) | OR (95% CI) | n | AHEI-2010, m (sd) | OR (95% CI) |
| Age-adjusted model 1 |  |  |  |  |  |  |  |  |  |
| AHEI-2010 tertile 1 | 290 | 38.5 (4.3) | 1.00 (ref) | 398 | 38.1 (4.7) | 1.00 (ref) | 207 | 38.2 (4.1) | 1.00 (ref) |
| AHEI-2010 tertile 2 | 303 | 47.4 (1.9) | 1.00 (ref) | 416 | 47.4 (2.0) | 0.99 (0.76-1.28) | 165 | 47.4 (2.1) | 0.72 (0.54-0.97) |
| AHEI-2010 tertile 3 | 262 | 56.3 (4.1) | 1.00 (ref) | 374 | 56.1 (4.4) | 1.00 (0.77-1.31) | 172 | 55.8 (4.2) | 0.82 (0.60-1.12) |
| P for trend |  |  |  |  |  | 0.98 |  |  | 0.18 |
| Multivariable-adjusted model 2# |  |  |  |  |  |  |  |  |  |
| AHEI-2010 tertile 1 | 207 | 38.5 (4.3) | 1.00 (ref) | 207 | 38.1 (4.7) | 1.00 (ref) | 207 | 38.2 (4.1) | 1.00 (ref) |
| AHEI-2010 tertile 2 | 165 | 47.4 (1.9) | 1.00 (ref) | 165 | 47.4 (2.0) | 0.98 (0.76-1.28) | 165 | 47.4 (2.1) | 0.72 (0.53-0.97) |
| AHEI-2010 tertile 3 | 172 | 56.3 (4.1) | 1.00 (ref) | 172 | 56.1 (4.4) | 1.02 (0.78-1.33) | 172 | 55.8 (4.2) | 0.86 (0.63-1.19) |
| P for trend |  |  |  |  |  | 0.91 |  |  | 0.32 |

P for trend were calculated using the quintile median values.

Multivariable-adjusted model 2 includes age, energy intake, physical activity, smoking, educational level, marital status and having farmer parents.

**eFigure 1. Associations of AHEI-2010, body mass index and the asthma symptom score using SEM**

AHEI-2010

Asthma symptom score

Body mass index

**Quintile 1** (ref)

**Quintile 2** MSR=0.92 (0.84-1.01) (p=0.09)

**Quintile 3** MSR=0.88 (0.80-0.96) (p=0.01)

**Quintile 4** MSR=0.90 (0.82-0.99) (p=0.02)

**Quintile 5** MSR=0.83 (0.75-0.91) (p<0.0001)

MSR=1.07 (1.06-1.08) (p<0.0001)

**Quintile 1** (ref)

**Quintile 2** β=-0.25 (-0.47;-0.03) (p=0.03)

**Quintile 3** β =-0.31 (-0.53;-0.09) (p=0.01)

**Quintile 4** β =-0.53 (-0.74;-0.29) (p<0.0001)

**Quintile 5** β =-0.74 (-0.96;-0.52) (p<0.0001)

Confounders

Confounders: age, energy intake, physical activity, smoking, educational level, marital status, having farmer parents. AHEI-2010 in quintiles, body mass index as a continuous variable, and the asthma symptom score as a count variable. β: difference in the expected BMI according to quintiles of healthy diet (ref: unhealthier diet). MSR: mean score ratio; a change in the asthma score is reported for an increase/decrease of 1 quintile in the AHEI-2010 diet score or 1 kg/m² in BMI.

AHEI-2010

Uncontrolled asthma

Body mass index

**Tertile 1** (ref)

**Tertile 2** OR=0.75 (0.56-1.00) p=0.05

**Tertile 3** OR=0.92 (0.68-1.23) p=0.56

OR=1.04 (1.0-1.06) p=0.01

**Tertile 1** (ref)

**Tertile 2** β=-0.19 (-0.69;0.32) p=0.46

**Tertile 3** β =-0.65 (-1.17;-0.14) p=0.01

Cofounders

Confounders: age, energy intake, physical activity, smoking, educational level, marital status, having farmer parents. AHEI-2010 in tertiles, body mass index as a continuous variable, and the asthma control as a binary variable. β: difference in the expected BMI according to tertiles of healthy diet (ref: unhealthier diet). OR: odds ratio; a change in the asthma score is reported for an increase/decrease of 1 tertile in the AHEI-2010 diet score or 1 kg/m² in BMI.

**eFigure 2.** **Associations of AHEI-2010, body mass index and uncontrolled asthma using SEM**
